# Ponatinib and other clinically approved inhibitors of Src and Rho-A kinases abrogate dengue virus serotype 2- induced endothelial permeability

**DOI:** 10.1080/21505594.2025.2489751

**Published:** 2025-04-06

**Authors:** Srishti Rajkumar Mishra, Ayan Modak, Mansi Awasthi, Archana Sobha, Easwaran Sreekumar

**Affiliations:** a Molecular Virology Laboratory, BRIC-Rajiv Gandhi Centre for Biotechnology (BRIC-RGCB), Thiruvananthapuram, India; b Regional Centre for Biotechnology (RCB), Faridabad, India; c Animal Research Facility, Rajiv Gandhi Centre for Biotechnology (RGCB), Thiruvananthapuram, India; d Molecular Bioassay Laboratory, Institute of Advanced Virology (IAV), Thonnakkal, Thiruvananthapuram, India

**Keywords:** Dengue virus, vascular leakage, platelets, TEER, AG129 mice

## Abstract

Severe dengue often presents as shock syndrome with enhanced vascular permeability and plasma leakage into tissue spaces. *In vitro* studies have documented the role of Src family kinases (SFKs) and RhoA-kinases (ROCK) in dengue virus serotype 2 (DENV2)-induced endothelial permeability. Here, we show that the FDA-approved SFK inhibitors Bosutinib, Vandetanib and Ponatinib, as well as the ROCK inhibitors, Netarsudil and Ripasudil significantly inhibit DENV2-induced endothelial permeability. In cultured telomerase immortalized human microvascular endothelial cells (HMEC-1), treatment with these inhibitors reduced the phosphorylation of VE-Cadherin, Src and myosin light chain 2 (MLC2) proteins that were upregulated during DENV2 infection. It also prevented the loss of VE-Cadherin from the inter-endothelial cell junctions induced by viral infection. In *in-vivo* studies using DENV2-infected AG129 IFN receptor-α/β/γ deficient mice, ponatinib, when administered 24 h post-infection onwards, demonstrated significant benefits in improving body weight, clinical outcomes, and survival rates. While all virus-infected, untreated mice died by day-10 post-infection, 80% of the ponatinib-treated mice survived, and approximately 60% were still alive at the end of the 15-day observation period. The treatment also significantly reduced disease severity factors such as vascular leakage, thrombocytopenia; mRNA transcript levels of proinflammatory cytokines such as IL-1β and TNF-α; and restored liver function. Comparable effects were observed even when ponatinib treatment was initiated after symptom onset. The results highlight ponatinib as an effective therapeutic option in severe dengue; and also a similar potential for other FDA- approved SFK and ROCK inhibitors.

## Introduction

Dengue has established as a major arboviral infection in the tropics [1]. While the disease remains a mild febrile illness in the majority of infected individuals, some develop severe dengue characterized by thrombocytopenia and/or transient shock syndrome [2,3]. Extreme thrombocytopenia or shock syndrome necessitates timely therapeutic intervention to prevent mortality. However, the current lack of effective drugs or specific treatment modalities against the disease necessitates the identification of newer pharmacological interventions to address the complications of Dengue.

Dengue is caused by any of the four serotypes of the dengue virus (DENV 1–4), a small, enveloped, positive-sense, single-stranded RNA virus belonging to the *Flaviviridae* family [4,5]. In infected patients, the disease may exhibit three distinct clinical phases of progression – febrile, critical and recovery [6]. While fever and viraemia characterize the febrile phase, thrombocytopenia and endothelial dysfunction resulting in plasma leakage and organ impairment are the hall marks of the critical phase, which occurs in a subset of patients post-defervescence and typically lasts for 1–2 days [2,6,7].

The major known contributors to endothelial dysfunction in dengue are inflammatory cytokines such as TNF-α, mast cells and their released angiogenic factors such as vascular endothelial growth factor (VEGF), circulatory mediators such as angiopoietin-2 (Ang-2) and other mediators like macrophage inhibitory factor (MIF); and the DENV NS1 protein [8–13]. Along with these inflammatory and vasoactive mediators, DENV has also been shown to directly infect endothelial cells *in vitro* contributing to the alteration of endothelial barrier function [3,7]. This perturbation of the barrier function occurs through the destabilization of inter-cellular junctions and the activation of actomyosin contractility within endothelial cells [3].

Regulation of microvascular endothelial permeability occurs at the cellular level via intracellular signalling events mediated by molecules that induce changes in the endothelial junctions as well as in transcellular protein transport [14]. For instance, the role of Src-family kinases (SFKs) in the modulation of upstream signalling that results in endothelial hyperpermeability has been demonstrated by various researchers [14–16]. Additionally, RhoA, an important member of the Rho family small GTPase proteins, and its downstream effector, Rho kinase (ROCK) are well known for their ability to modulate actin organization involved in the regulation of barrier function [17,18]. Stimulation by VEGF is regulated by SFK-Src; where the receptor VEGFR2-mediates Src/Rac/Pak signalling, which subsequently leads to VE-Cadherin internalization [16,19]. Similar responses have been demonstrated in VEGF/histamine-induced RhoA-ROCK-MLC activation via actin stress formation [20,21]; these pathways ultimately enhance vascular permeability.

DENV infection results in increased vascular permeability; however, the molecular processes and signalling events involved in the endothelial cells are poorly understood. Previous studies have shown that Src kinase inhibitors suppressed Andes virus-induced endothelial hyperpermeability [16]. In 2018, Soliman et al showed that inhibition of the RhoA/ROCK pathway prevent tight junction permeability induced by rotavirus infection [22]. In our previous studies, we demonstrated that similar mechanisms were involved in dengue virus serotype 2 (DENV2) infection. We found a significant increase in Src/RhoA phosphorylation, ultimately augmenting the phosphorylation of VE-Cadherin, with enhanced permeability of DENV2-infected telomerase-immortalized human microvascular endothelial cells (HMEC-1) monolayer [13]. Moreover, the Rho-kinase inhibitor Y-27632 and Src-kinase inhibitor, dasatinib restored the increased permeability [13]. These observations prompted us to conduct additional experiments on Src Family kinase (SFK) and Rho kinase (ROCK) inhibitors in modulating heightened endothelial permeability following DENV2 infection.

In this study, we evaluated the efficacy of five FDA-approved small molecule inhibitors of SFK (Bosutinib, Vandetanib, and Ponatinib), and ROCK (Netarsudil and Ripasudil) in reducing DENV2-induced permeability in cultured HMEC-1 monolayers. Using *in vivo* experiments, we also examined the ability of the SFK inhibitor, ponatinib to mitigate vascular permeability in DENV2- infected AG129 mice.

## Materials and method

### Cells and virus

HMEC-1 (Telomerase Immortalized Human Dermal Microvascular Endothelial Cell), was acquired from ATCC (CRL 4025) and cultured in F-12K (Invitrogen, USA) medium supplemented with growth factors FGF (fibronectin growth factor, Invitrogen), EGF (epidermal growth factor, Invitrogen), heparin (Sigma Aldrich), 20% (v/v) heat-inactivated Foetal bovine serum (FBS, Gibco) and antibiotic-antimycotic mix (ABM, Sigma Aldrich). BHK-21 (ATCC CCL-10) and C6/36 mosquito cell lines were grown in DMEM and L-15 medium respectively, supplemented with 10% (v/v) heat-inactivated FBS and 1X antibiotic-antimycotic cocktail. DENV2 strain RGCB880/KL/2010 (GenBank Accession number KY427084), which was isolated in our laboratory from the serum of a 45-day-old female individual with severe dengue was used in the study [23]. The virus was passaged in the C6/36 cell line and used in *in vitro* experiments. For mock infection, heat-inactivated RGCB880/KL/2010 DENV2 virus (inactivated at 56°C for 60 mins) was used. The virus was quantified by titrating in BHK-21 cells by plaque assay as previously described [13,24].

### Drugs (SFK and ROCK inhibitors)

The Src-family kinase inhibitors- bosutinib {SKI-606; 4-[(2,4-dichloro-5-methoxy-phenyl) amino]-6-methoxy-7-[3-(4-methylpiperazin-1-yl) propoxy] quinoline-3-carbonitrile}, vandetanib {ZD6474; *N*-(4-bromo-2-fluorophenyl)-6-methoxy-7-[(1-methylpiperidin-4-yl) methoxy] quinazolin-4-amine}, ponatinib {AP24534; 3-(2-{imidazo [1,2-b] pyridazin-3-yl} ethynyl)-4-methyl-N-{4-[(4-methylpiperazin-1-yl)methyl]-3 (trifluoromethyl) phenyl} benzamide; and the Rho-kinase inhibitors- netarsudil {AR-13324; [4-[(2S)-3-amino-1-(isoquinolin-6-ylamino)-1-oxopropan-2-yl] phenyl] methyl 2,4-dimethylbenzoate}, ripasudil, (as hydrochloridehydrate) {K-115; 4-fluoro-5-[[(2S)-2-methyl-1,4-diazepan-1-yl] sulfonyl] isoquinoline} were purchased from Selleck Chemicals and dissolved in Dimethyl sulfoxide (DMSO) or water according to the manufacturers’ instructions.

### In vitro trans-endothelial permeability measurement

The *in vitro* permeability assay was carried out according to previously described protocols with minor modifications as indicated [13,24]. Polyethylene Terephthalate (PET), semi-permeable trans-well inserts (0.4 µm pore size, 6.5 mm diameter; CLS3470; Corning Costar) were coated with collagen and fibronectin; HMEC-1 cells were then seeded on these dual coated inserts placed in its compatible 24-well culture plate. The cells were allowed to grow until a confluent monolayer was formed. These cells were infected with DENV2 or heat-inactivated virus (for mock-infection) at an MOI of 5. At 2 h post-infection, the inoculums were removed and replaced with fresh F12K medium containing 5% FBS with or without the drugs mentioned previously, at their respective nanomolar concentrations. The alterations in endothelial permeability, *in vitro*, were initially measured by a trans-endothelial electrical resistance (TEER) assay using an EndOhm-6 chamber and an EVOM voltmeter (World Precision Instruments, USA). The TEER across the monolayer was measured every 12 h using dipping and bottom electrodes provided along with the instrument. Further, permeability changes were also measured using the FITC-dextran permeation assay, where 1 mg/mL of FITC-dextran (70 kDa, FD70S, Sigma-Aldrich) in Hank’s balanced salt solution (HBSS) (1X) (14025-076; Gibco) was added into the upper chamber of the trans-well culture plate, at 36 h post-infection. After 1 h, the medium was collected from the lower chamber, and the fluorescence intensity was measured using a fluorescence reader (485 nm excitation; 535 nm emission). Dextran permeability was demonstrated as an increase in the percentage of FITC-dextran level over the basal permeability seen in the mock-infected monolayer at 36 h post-infection.

### Immunoblotting

Whole cell lysates from HMEC-1 cells and liver tissue extracts from mice were prepared in radio-immunoprecipitation assay (RIPA) lysis buffer (SIGMA; USA) supplemented with a protease inhibitor and phosphatase inhibitor cocktail (SIGMA; USA). After lysis, the supernatant was collected by centrifugation; and the protein concentration was determined using Bradford’s assay. Protein denaturation was carried out by boiling it in 5X sample buffer for 5 min. Equal amounts of protein were resolved by 10% or 15% SDS-PAGE and transferred onto polyvinylidene fluoride membranes (Amersham, Hybond, Germany). The membranes were blocked with 5% bovine serum albumin (BSA) in Tris-Buffered saline with Tween-20 (TBST) for 1 h, on a rocker at room temperature. After washing twice for 5 min each the membranes were incubated with primary antibodies against target proteins at 4°C for overnight. The following day, the membranes were washed with TBST four times for 10 min each; and then incubated with secondary antibodies conjugated with horse radish peroxidase (details of primary and secondary antibodies are given in the Supplementary Table S1) for 1 h at room temperature on a rocker. The membranes were then washed with TBST four times for 10 min each; and the bands were visualized using an enhanced chemiluminescence (ECL) kit (Takara). The intensity of signal was quantified using ImageJ software (National Institutes of Health, USA). The band intensities of target proteins were normalized to the intensities of β-actin and the ratio of phosphorylated proteins to the respective total proteins was plotted.

### Immunofluorescence assay

Immunofluorescence assays were performed according to a previously described protocol [24]. In brief, HMEC-1 cells were grown until confluent monolayer was formed on the cover slips. The monolayer was then infected with DENV2 or heat-inactivated virus (mock infection), followed by the treatment with ponatinib and incubation for 36 h. Cells were fixed with 4% formaldehyde for 20 min and permeabilized with 0.1% Triton X-100. Cells were blocked with 5% bovine serum albumin (BSA) in PBS for 1 h at room temperature, followed by three washes with PBS. The coverslips were then incubated overnight at 4°C with primary antibodies against VE-Cadherin and dengue virus E glycoprotein. The following day, the coverslips were washed with PBS for three times and incubated with secondary antibodies) for 1 h at room temperature. Cell nuclei was counterstained with DAPI (1 mg/ml). Final washes were performed with PBS, and the coverslips were mounted on clean microscopy slides. Images were captured and viewed under a NIKON A1R confocal microscope. (Details of the antibodies are given in Supplementary Table S1)

### Ethics statement and DENV2-infection animal model

All animal experiments were carried out with the approval of the BRIC-Rajiv Gandhi Centre for Biotechnology (BRIC-RGCB) Institutional Animal Ethics Committee (IAEC) (IAEC/742/SREE/2019). AG129 mice (interferon receptor (α/β/γ) deficient strain), were purchased from B&K Universal (UK) and were then housed, bred and maintained in specific pathogen-free settings. AG129 mice (3–4 weeks old; 8–9 g weight) were maintained in individually ventilated cages (3–4 animals per cage) with *ad libitum* feed and water. They were infected as described previously with a 35°C-passaged (35P) RGCB880/KL/2010 DENV2 virus strain via the subcutaneous route (s/c; 10^4^ pfu/mice in 500 µL) [24,25]. For mock infection, an equal volume of heat-inactivated virus was used.

### Experimental design

#### Pre-symptom-onset treatment regimen

To study the effect of the SFK inhibitor ponatinib on parameters *in vivo*, AG129 mice were randomly divided into the following four groups: (1) mock infection group (mice injected with heat-inactivated virus), (2) ponatinib alone group (mice that received ponatinib alone), (3) DENV2-infection group (mice injected with RGCB880 35P DENV2 virus; and (4) DENV2+Ponatinib (mice injected with DENV2 followed by the Ponatinib Treatment). The drug treatment was started on day 1 post-infection at a dose 15 mg/kg/day [26], considering the day of infection as day 0, and continued until 15 days post-infection.

#### Post-symptom-onset treatment regimen

In this set of experiments, along with ponatinib, we also used the drug FTY720 (Fingolimod) which was found to be beneficial in our earlier studies in alleviating vascular permeability in DENV-infected AG129 mice [24]. The mice were divided into following six groups: (1) mock infection group; (2) DENV2-infection group; (3) Ponatinib + FTY720 group (mice that received a combination of ponatinib 15 mg/kg/day and FTY720 5 mg/kg/day); (4) DENV2+Ponatinib treatment group; (5) DENV2+FTY720 treatment group; and (6) DENV2+Ponatinib + FTY720 (DENV2 infection followed by mice that received combination of ponatinib 15 mg/kg/day and FTY720 5 mg/kg/day). Drug treatment was started at Day-4 post-infection (when symptoms started developing) and continued until 15 days post-infection.

### In vivo permeability assay

To assess vascular barrier function, Evans blue assay (Miles’ assay) was carried out according to a previously published protocol [24,25]. Briefly, AG129 mice were subcutaneously infected with DENV2 (10^4^ pfu) or left uninfected as a control, followed by treatment with ponatinib. These mice were then injected under isoflurane anaesthesia with 100 µl of Evans Blue dye (0.5%) intravenously (tail vein route) on the 6^th^ or 7^th^ day post-infection, when they were at the peak of the symptoms. After two hours, the mice were euthanized and extensively perfused with sterile 1X PBS in order to wash out any dye within the vasculature. The organs were harvested and oven dried overnight at 55 °C. They were weighed, suspended in 500 μL formamide in individual tubes and incubated at 55 °C in a water bath for 48 h. The concentration of the dye extracted from the tissues was determined spectrophotometrically at an absorbance of 610 nm and compared with a standard curve of known quantities of Evan ‘s blue dye.

### Haematology and blood biochemistry

On 7^th^ day post-infection, blood was collected from the mice by cardiac puncture in ethylene diamine tetra acetic acid (EDTA) tubes (K2 EDTA, Cat No. 367838, BD) and plain micro centrifuge tubes. Whole blood samples were analysed for haematological parameters using an automated analyser (Nihon Kohden MEK 6550); and serum samples were analysed for biochemical parameters according to the manufacturer’s instructions (Fujifilm DRI-CHEM NX500i)

### Quantification of DENV2 RNA copy number

Isolation viral RNA and quantification of DENV2 RNA copies were performed according to previously published protocols [24,25]. Whole blood samples were collected from DENV2-infected and mock-infected mice, and the total RNA isolated was subjected to real-time RT-PCR. The copy numbers of viral RNA were calculated as described previously [25].

### Immunohistochemistry

Liver tissues harvested from sacrificed mice were fixed in 10% neutral buffered formalin (NBF) and embedded in paraffin. Immunohistochemistry was performed using the PolyExcel HRP/DAB Detection System according to the manufacturer’s protocol (PEH002, Pathnsitu, USA). Briefly, these tissues were sectioned, dewaxed, rehydrated in xylene and a series of alcohol. Antigen retrieval was performed in sodium citrate buffer (pH = 6.0) by boiling and endogenous peroxidases were blocked with hydrogen peroxide for 10 min. The sections were incubated overnight with primary antibodies against VE-Cadherin; and developed using DAB as a substrate and counterstained with haematoxylin (Himedia, India). The stained tissue sections were examined under a fluorescence microscope (Zeiss, Göttingen, Germany); and 3–4 independent fields were imaged (20×) for each group.

### Statistical analysis

Student’s t-test (Unpaired) or One-way ANOVA was performed using Graph Pad Prism 8 software. All experiments were independently repeated three times, unless indicated otherwise. Statistical significance was represented as *p* < 0.05 (*p < 0.05, **p < 0.01, ***p < 0.001, **** < 0.0001).

## Results

### Rho-kinase (ROCK) inhibition abrogates DENV2-induced endothelial permeability and reduces myosin light chain 2 (MLC2) and VE-Cadherin phosphorylation in HMEC-1 cells

**Figure 1.**
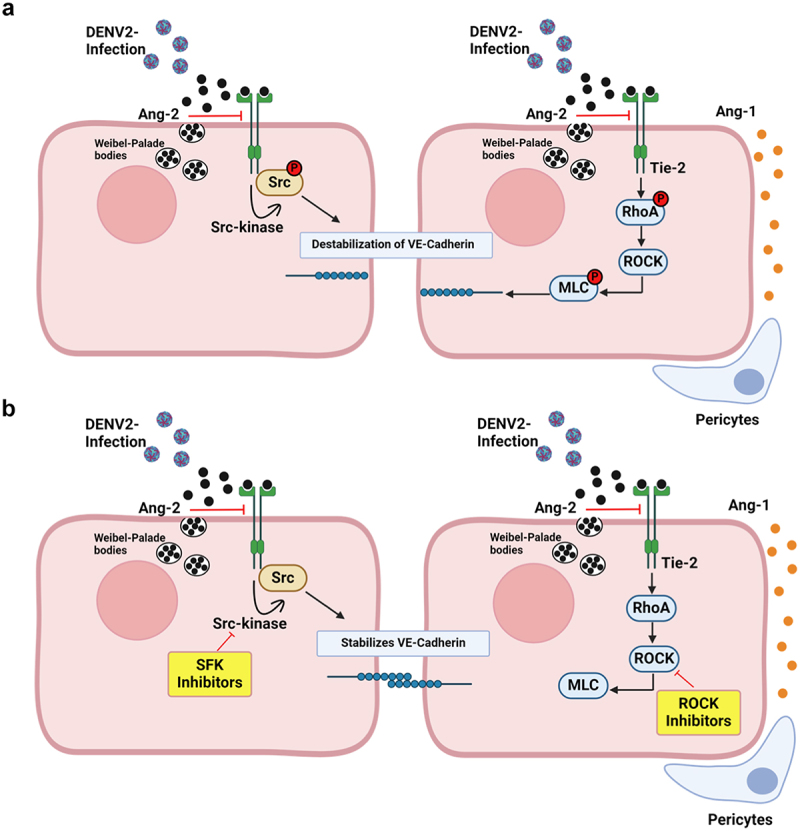
Targeting Angiopoietin/Tie-2 pathway to prevent dengue virus-induced endothelial permeability: In physiological conditions, endothelial junction integrity is maintained by Ang/Tie 2 pathway through the stabilization of VE -Cadherin junctions by Ang-1 acting as an agonist and signalling through the Tie2 receptor. (a). In disease conditions like DENV infection, mediators activate the release of Ang-2 by Weibel Palade bodies which act as an antagonist leading endothelial permeability. The signalling cascade is mediated through sequential phosphorylation of Tie2-Src, RhoA/ROCK and VE- Cadherin proteins and internalization of VE-Cadherin. (b). Upon treatment with SFK and ROCK inhibitors, the enhanced endothelial permeability is reversed by preventing the VE-Cadherin phosphorylation and its internalization.

**Figure 2.**
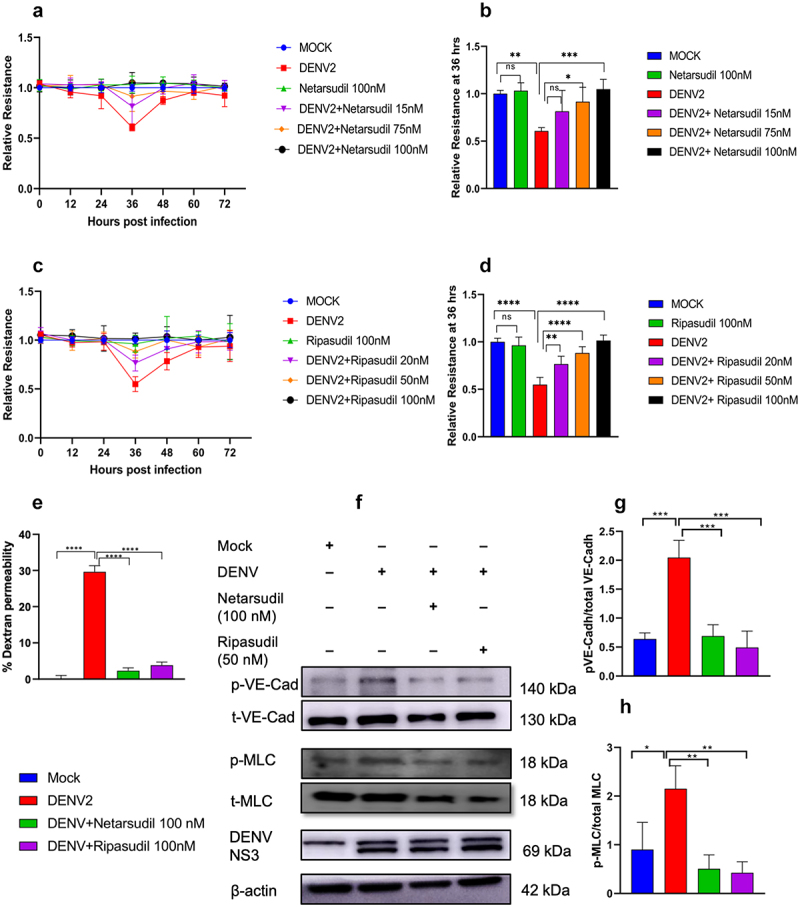
Rho-kinase (ROCK) inhibition abrogates DENV2-induced endothelial permeability and reduces MLC2 and VE-Cadherin phosphorylation in HMEC-1 cells. HMEC-1 cells were infected with DENV2 (RGCB880 strain) at a MOI of 5 followed by treatment with ROCK inhibitors- Netarsudil and Ripasudil. (a & c) Trans-endothelial electrical resistance (TEER) kinetics analysis: the kinetics of endothelial permeability were evaluated using the TEER assay at every 12 h. (b & d) Relative TEER resistance quantification done at 36 h post-infection. The data are mean of two independent experiments each with duplicate at each time point (*n* = 4). (e) FITC-Dextran assay: the permeability of endothelial monolayers was assessed at 36 h post-infection with FITC-dextran. Mean values from three independent experiments, each with duplicate (*n* = 6) readings are shown. Statistical significance was determined by one way-anova with Sidak’s multiple comparisons test. (f) Western blot analysis: expression levels of p-VE-Cadherin, VE-Cadherin, p-MLC2, MLC2 along with DENV2-NS3 were assessed in HMEC-1 cells following either DENV2 infection, MOCK infection (heat-inactivated DENV2) or with Netarsudil and Ripasudil treatment (100 nM) at 36 h post-infection. (g-h) Densitometric analysis of the band intensities in western blot for p-VE-Cadherin/VE-Cadherin (g) and p-MLC2/MLC2 (H). Data are presented as mean ± SD from three independent experiments. Statistical analysis was done by one-way ANOVA with Sidak’s multiple comparisons test. Significance is denoted by “*” (*p* < 0.05), “**” (*p* < 0.005), “***” (*p* < 0.0005), “****” (*p* < 0.00005), and “ns” for non-significant results.

In our previous studies we demonstrated that Src and RhoA associated kinases (ROCK) have the potential to inhibit DENV2-induced permeability *in vitro* in HMEC-1 cells [13]. Time-kinetics analysis of DENV2 infection in HMEC-1 cells indicated maximum infection by 36–48 hours post-infection (Supplementary Figure S2b). Similarly, in our TEER assay results, maximum permeability was observed at 36 h post-infection (Supplementary Figure S2a). Based on this observation, we went ahead to check the hypothesis whether inhibiting these molecules can alleviate endothelial permeability changes induced by DENV2 infection at 36 h post-infection (Figure 1). It is known that the drugs Netarsudil and Ripasudil block ROCK activity [27,28]. Initially we checked the effect of these drugs on cell viability in HMEC-1 cells and MTT assay results confirmed that both Netarsudil and Ripasudil were not toxic even at concentrations up to 100 nM (Supplementary Figure S1b). Further, using the TEER assay, we assessed the ability of these ROCK inhibitors to block DENV2-induced permeability in HMEC-1 cell monolayers grown on Transwell plates. DENV2-induced permeability was inhibited by both Netarsudil and Ripasudil in a dose-dependent manner with the maximum inhibition observed at 100 nM (Figure 2(a-d)). In addition, the FITC-Dextran permeability assay performed at 36 h post-infection showed a significant reduction in monolayer permeability in ROCK inhibitor-treated cells, confirming the restoration of endothelial barrier function (Figure 2(e)). It was also observed in western blot experiments that there is a normalization in the phosphorylation status of VE-Cadherin and myosin light chain 2 (MLC2), which are the downstream targets of Rho/ROCK signalling, in DENV-2 infected HMEC-1 cells upon treatment with these inhibitors (Figure 2(f-h)).

### Inhibition of Src-kinase attenuates DENV2-induced hyperpermeability and reduces Src and VE-Cadherin phosphorylation in HMEC-1 cells

**Figure 3.**
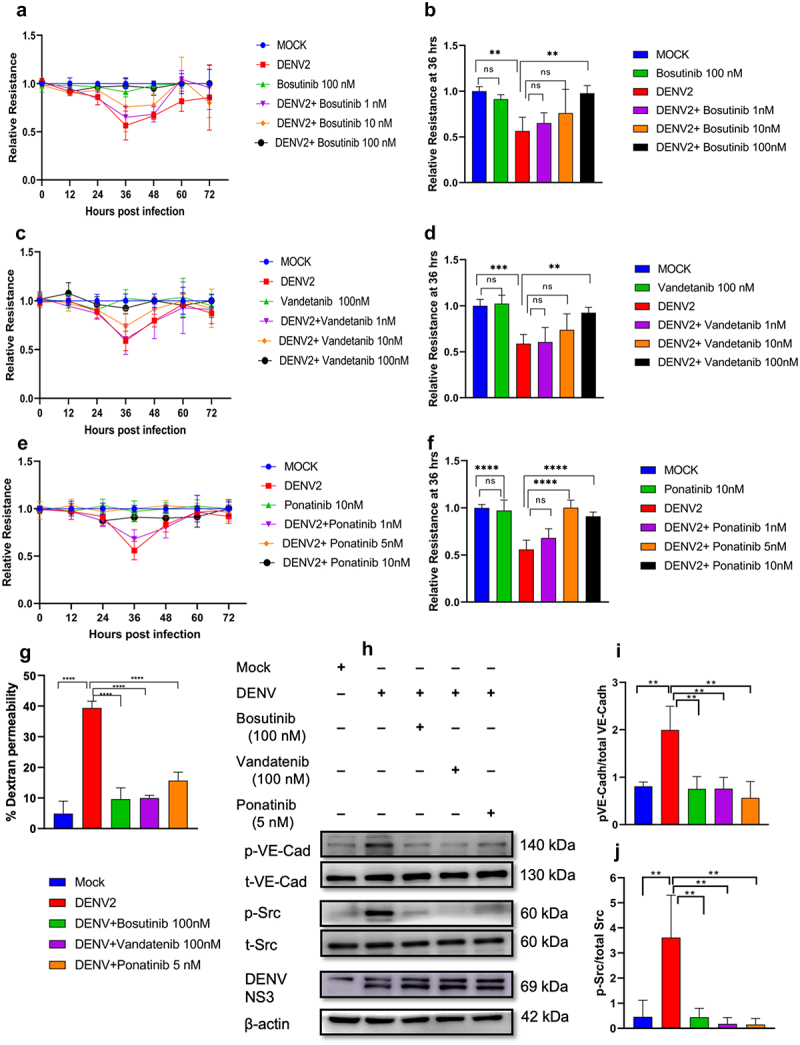
Inhibition of Src-kinase attenuates DENV2-induced hyperpermeability and reduces Src and VE-Cadherin phosphorylation in HMEC-1 cells. (a, c & e) Changes in trans-endothelial permeability upon treatment with FDA-approved SFK inhibitors Bosutinib, Vandetanib and Ponatinib upon DENV2 infection were assessed using the TEER assay at every 12 h. (b, d & f) Relative TEER quantification done at 36 h post-infection. Mean values from two independent experiments each with duplicate readings at each time point (n = 4) (g) FITC-dextran assays at 36 h post-infection in DENV2-infected HMEC-1 cells. Mean values from three independent experiments, each with duplicate (n = 6) readings. Statistical significance was determined by one-way ANOVA with Sidak’s multiple comparisons test. (h) Western blot analysis to detect levels of p-VE-Cadherin, VE-Cadherin, *p*-Src, Src, and NS3 in cells infected with DENV2 or MOCK infected; and treated with Bosutinib (100 nM), Vandetanib (100 nm) and Ponatinib (5 nM) at 36 h p.I. (i & j) Densitometric analysis of the band intensities in western blot for p-VE-Cadherin/VE-Cadherin and *p*-Src/Src. Results are expressed as mean ± SD from three experiments, with statistical significance (one-way ANOVA with Sidak’s multiple comparisons test); and indicated as “*” (p < 0.05, “**” (p < 0.005), “***” (p < 0.0005), “****” (p < 0.00005), and “ns” signifies no statistical significance.

Since the modulation of Src-kinase upon DENV2 infection in HMEC-1 cells was also observed in our previous studies [13], we further examined the effect of its inhibition using the SFK inhibitors Bosutinib, Vandetanib and Ponatinib. Results from the MTT assay indicated that these inhibitors had no toxic effects in HMEC-1 cells (Supplementary Figure S1a). At these non-cytotoxic concentrations, SFK inhibitors demonstrated efficacy in alleviating DENV2-induced endothelial permeability (Figure 3) at 36 h post-infection. TEER data indicated that 100 nM bosutinib and vandetanib offered maximum reduction in barrier disruption (Figure 3(a-d)). On the other hand, ponatinib, was able to bring about a comparable reduction at concentrations of 5 nM and 10 nM (Figure 3(e,f)). The FITC-Dextran permeability assay results corroborated these observations demonstrating a complete blockade of trans-endothelial leakage 36 h post-infection at the most effective drug concentrations (Figure 3(g)). Similar to the observations made in the treatment with ROCK-inhibitors, treatment with SFK inhibitors such as bosutinib, vandetanib, and ponatinib at their maximum effective concentrations resulted in complete alleviation of Src and VE-Cadherin phosphorylation at 36 h post-infection (Figure 3(h-j)).

These findings demonstrate that at nanomolar concentrations SFK and ROCK inhibitors showed comparable responses in restoring barrier function. Ponatinib showed efficacy in reducing microvascular permeability at drug concentrations as low as 5 nM and, hence, further experiments were carried out using it.

### Treatment with Ponatinib restores VE-Cadherin junctions in DENV2-infected HMEC-1 cells

**Figure 4.**
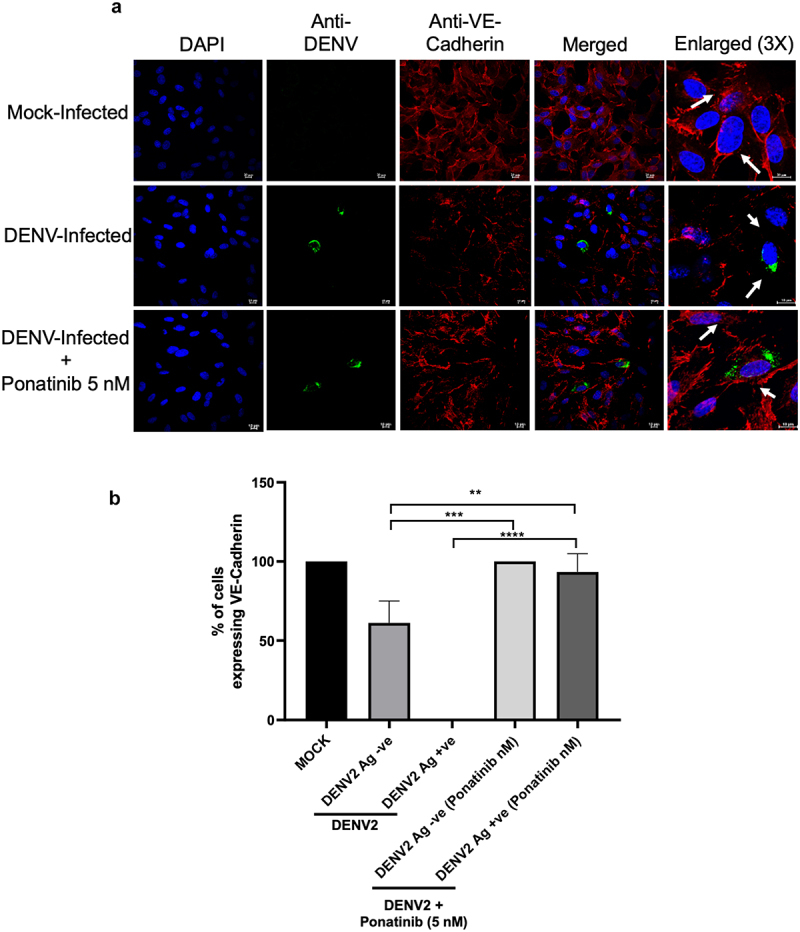
Treatment with Ponatinib reinstates VE-Cadherin expression at inter-cellular junctions in DENV2-infected HMEC-1 cells: (a) immunostaining: cells were co-immuno-stained for VE-Cadherin (red) and DENV2 (green) at 36 h post-infection. (b) To assess the VE-Cadherin expression, the proportion of cells with intact staining was quantified in both MOCK and DENV2-infected conditions. Counts of cells with intact VE-Cadherin were taken from three independent fields and represented as a percentage of the total number of dapi-stained cells. Statistical analysis was done using one-way ANOVA with Sidak’s multiple comparisons test, “**” for *p* < 0.005 and “****” for *p* < 0.00005 and “ns” for non-significance.

VE-Cadherin is a junctional protein that plays a critical role in maintaining the integrity of the vascular endothelial monolayers. In our previous study, we observed a decrease in VE-Cadherin expression in DENV2-infected HMEC-1 cells [13,29]. In the present study also, in DENV-infected cells, immunofluorescence staining at 36 h p.i showed significant reduction in the membrane expression of VE-Cadherin; and the treatment with Ponatinib restored its expression at the cellular junctions (Figure 4).

### Pre-symptom onset Ponatinib administration sustains body weight and improves survival in DENV2-infected AG129 mice

**Figure 5.**
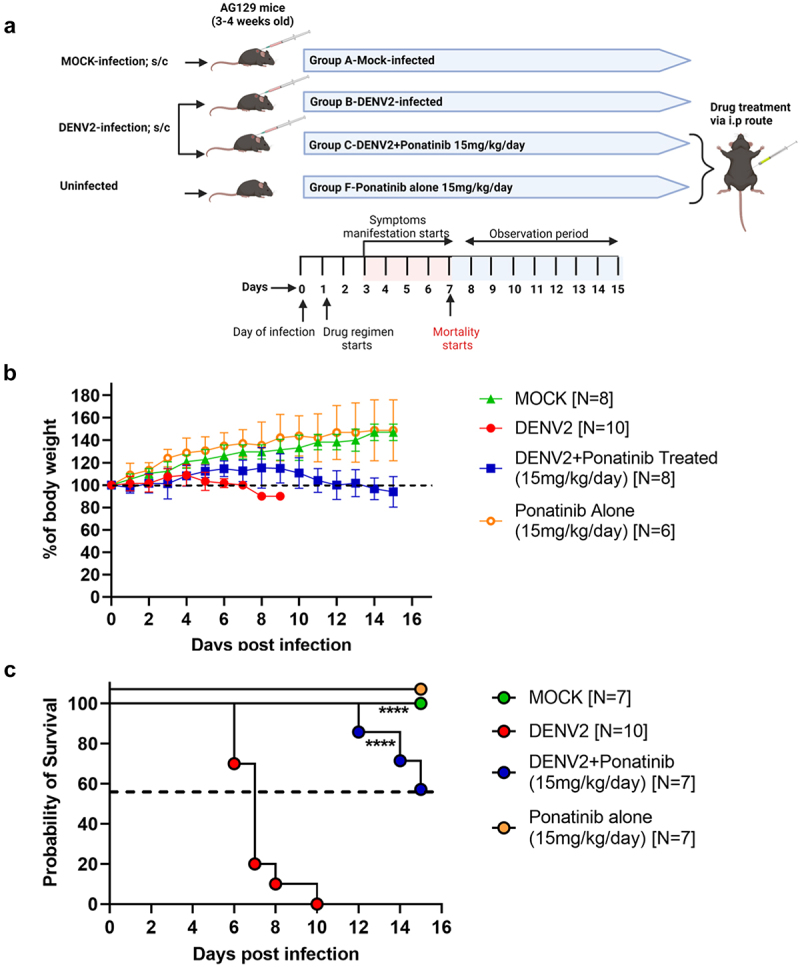
Pre-symptom onset treatment with Ponatinib maintains body weight and improves survival in AG129 mice infected with DENV (a) experimental scheme (b) body weight changes expressed as a percentage from the baseline measurement on day 0. (c) Comparative analysis of mortality rates among denv-infected, mock-infected, and Ponatinib-treated mice. Data shown are mean ± SD from 6 to 10 mice per group (denoted as “N”). The Log-rank (Mantel-Cox) test was used to determine the significance of survival differences based on median survival times.

A lethal model for dengue in AG129 mice infected with DENV2 serotype that shows haemorrhage, vascular leakage and mortality, with approximately 80% of infected mice succumbing within 7 days of infection, was established previously [24,25] Using this model, we observed that sub-cutaneous infection with 10^4^ PFU of DENV2 RGCB880 35P strain results in significant weight loss followed by severe symptoms (ruffled fur, hunched back posture, oedema, haemorrhagic manifestations and mortality) (Figure 5). In the pre-symptom onset treatment regimen, ponatinib was administered at a dose of 15 mg/kg/day from 1^st^ day post-infection (Figure 5(a)). In infected mice, treatment with ponatinib significantly improved body weight maintenance compared to that in untreated mice (Figure 5(b)). It also improves the survival rate. While all untreated, virus-infected mice died by day 10 post-infection, 100% of ponatinib treated mice were alive at 10th day, 80% on day 12; and 60% at the end of the 15-day observation period (Figure 5(c)).

### Pre-symptom onset Ponatinib treatment reduces vascular leakage in DENV2-infected mice

**Figure 6.**
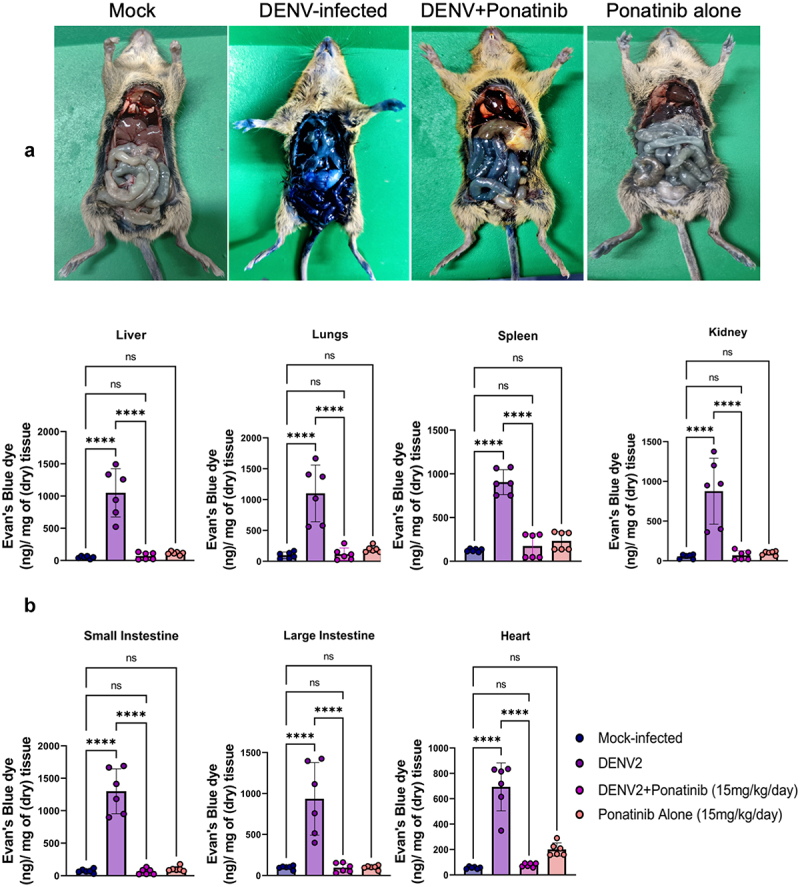
Pre-symptom onset treatment with Ponatinib reduces vascular leakage induced by DENV infection in AG129 mice. Mile’s permeability assay using Evan’s blue dye was performed as mentioned in the methodology. AG129 mice were either infected subcutaneously with 10^4^ pfu of DENV or kept as mock-infected controls. (a) Gross pathological images of vascular leakage with dye extravasation in the viscera of denv-infected mice along with visual changes observed upon Ponatinib treatment. (b) Quantitative analysis for Evan’s blue dye in tissues. Each point corresponds to the value from an individual animal. Results are displayed as mean ± SD from 6 mice per group. One-way ANOVA with Bonferroni’s multiple comparison test was used for statistical evaluation, with significance denoted as, “*” (*p* < 0.05, “**” (*p* < 0.005), “***” (*p* < 0.0005), “****” (*p* < 0.00005), and “ns” for not significant.

To assess the effect of ponatinib on vascular permeability in DENV2-infected mice, we performed a Mile’s assay using Evan’s blue dye. We observed a substantial increase in vascular leakage in DENV2-infected AG129 mice whereas administration of ponatinib at a dose of 15 mg/kg/day significantly reduced DENV2-induced vascular leakage, as evidenced by a visible reduction in the extravasated dye into the internal organs (Figure 6(a)). The quantitative analysis of the dye in various tissues confirmed these observations. A higher dye concentration was observed in the organs of virus-infected mice, which was significantly reduced in mice treated with ponatinib (Figure 6(b)). In control experiments, uninfected mice treated with Ponatinib showed no significant changes in vascular permeability, indicating that the drug did not induce any vascular permeability alterations (Figure 6(b)).

### Hematological parameters altered upon DENV2 infection in mice are improved on early treatment with Ponatinib

**Figure 7.**
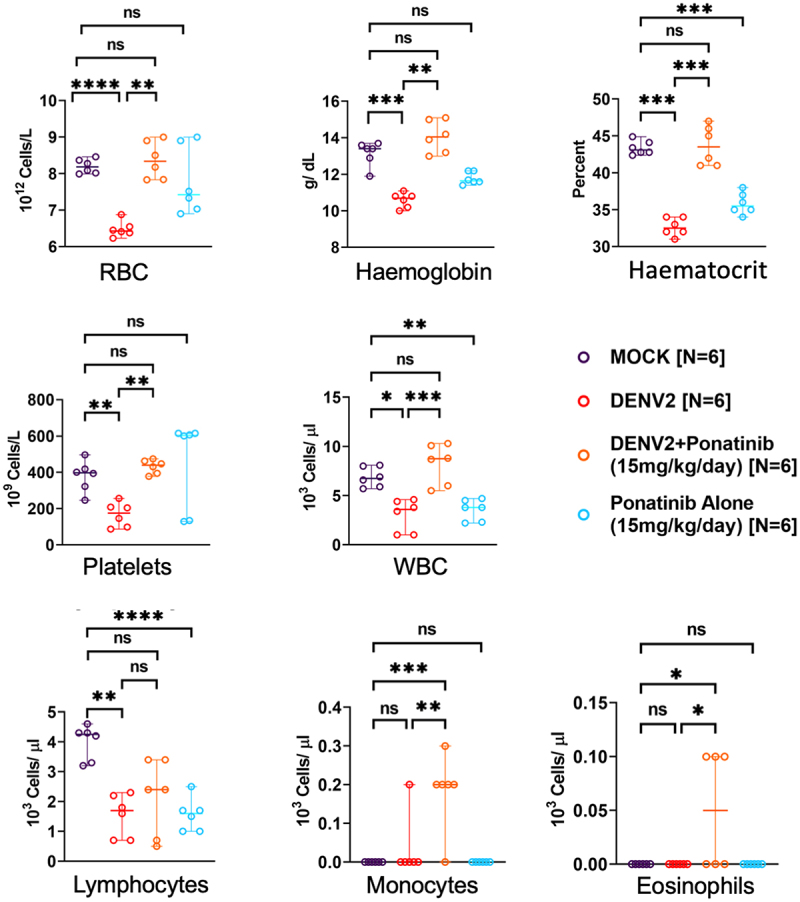
Pre-symptom onset treatment with Ponatinib improves the haemogram changes induced by DENV infection in AG129 mice.: mice were infected subcutaneously with 10^4^ pfu of DENV-2, or were mock-infected. On day 7 post-infection, when the disease was at its peak, blood samples were collected and analysed using an automated analyser (Nihon Kohden MEK 6550) for haematological parameters. Each point on the graph represents value from an individual mouse. Results are shown as mean ± SD from 6 to 7 mice per group (denoted as “N”). Statistical significance was determined using one-way ANOVA with Sidak’s multiple comparisons test., with significance levels indicated as “*” (*p* < 0.05, “**” (*p* < 0.005), “***” (*p* < 0.0005), “****” (*p* < 0.00005), and “ns” for not significant.

As observed in our earlier studies [24], DENV2 infection in mice caused erythrocytopenia, leucopenia, lymphopenia and thrombocytopenia when assessed on the 7^th^ day post-infection, at the peak of symptoms (Figure 7). There was also a significant decrease in haemoglobin and haematocrit, indicating blood loss due to haemorrhagic manifestations in this disease model. Pre-symptom onset treatment with ponatinib restored the cell count to normal levels, except for lymphocyte counts. It was observed that the ponatinib treatment itself caused a reduction in haematocrit values, but this was not as pronounced as due to the DENV2-infection. It also caused a reduction in the total leukocyte and lymphocyte counts compared to the mock-infected, untreated animals (Figure 7).

### Ponatinib treatment prevents alteration in liver enzyme levels in DENV2-infected mice

**Figure 8.**
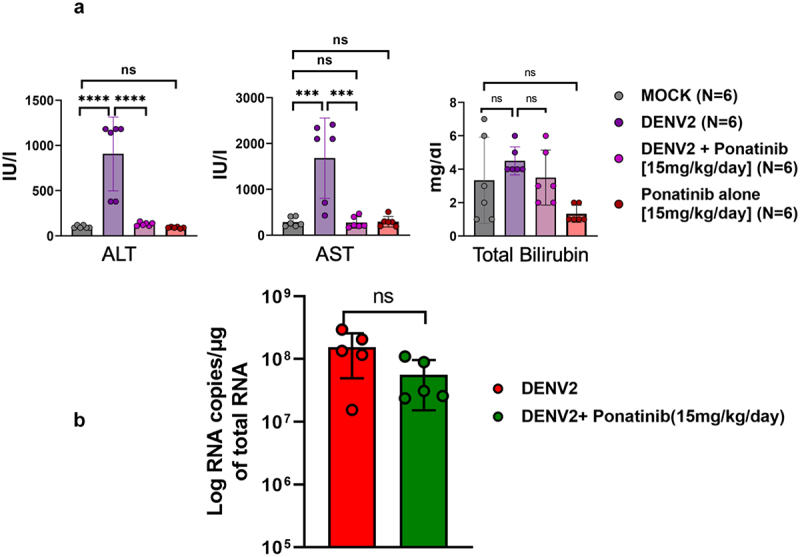
Pre-symptom onset treatment with Ponatinib improves liver function altered upon DENV2 infection in AG129 mice: (a) Mice were infected with 10^4^ pfu of DENV, subcutaneously, or remained mock-infected. On day 7 post-infection (peak of symptoms), blood samples from mice were collected and serum was analysed in an automated analyser (Fujifilm DRI-CHEM NX500i) for the parameters shown. Each data point denotes value from an individual mouse. Results are shown as mean ± SD from 6 mice per group (denoted as “N”). (b) Quantification of viral load in blood by real-time RT-PCR in order to assess antiviral effect of Ponatinib on DENV2- infected mice. Results are shown as mean ± SD from 5 mice per group. Statistical analysis was determined using one-way ANOVA with Bonferroni’s multiple comparison test, “*” (*p* < 0.05, “**” (*p* < 0.005), “***” (*p* < 0.0005), “****” (*p* < 0.00005), and “ns” signifies no statistical significance.

Hepatic dysfunction is one of the prominent signs of severe dengue; and in the DENV-infected mice, the serum levels of liver enzymes, aspartate aminotransferase (AST) and alanine aminotransferase (ALT), were significantly elevated (Figure 8(a)). However, there was no significant change in bilirubin levels after DENV infection. Ponatinib treatment started from 1^st^ day post-infection prevented the increase in the levels of AST and ALT (Figure 8(a)). Since the liver is one of the major sites of DENV replication, any possible antiviral effect mediated by ponatinib can also prevent tissue damage and elevation of enzyme levels. However, we observed that the viral load (RNA copies) in the whole blood samples was comparable at the peak of the symptoms (7th-day post-infection) in untreated and ponatinib-treated, DENV2-infected mice (Figure 8(b)). These results were in line with the observations made in *in vitro* cultures based on the plaque assay (Supplementary Figure S1c) suggesting that that ponatinib did not exert any direct antiviral activity against DENV2.

### Ponatinib treatment reduces the levels of phosphorylated Src and VE-Cadherin; and reinstates the VE-Cadherin expression in the liver tissues of DENV2- infected mice

**Figure 9.**
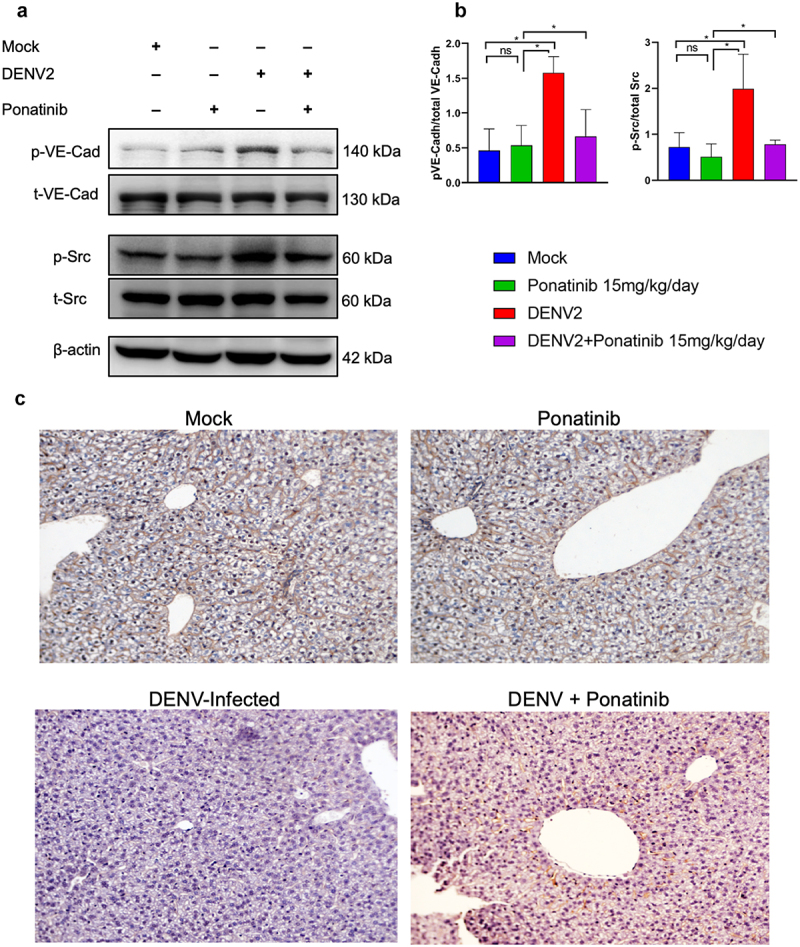
Pre-symptom onset treatment with Ponatinib reduces Src and VE-Cadherin phosphorylation and restores the sinusoidal VE-Cadherin expression in liver tissues of DENV2 infected AG129 mice: mice were either mock infected or infected with 10^4^ pfu of DENV2 followed by early treatment with Ponatinib, intraperitoneally (i. p) at 15 mg/kg/day dosage. (a) Western blot analysis of total protein from liver tissues to detect VE-Cadherin and Src levels. (b) Quantitative analysis of the expression levels of p-VE-Cadherin and *p*-Src. The ratio of phosphorylated proteins with corresponding total protein were plotted after normalizing with the expression levels of β-actin. Data are presented as mean ± SD from three independent experiments analysed withOne-way ANOVA with Sidak’s multiple comparisons test. Significance is denoted by “*” (p < 0.05, “**” (p < 0.005), “***” (p < 0.0005), “****” (p < 0.00005), and “ns” for non-significant results. (C) Immunohistochemical (IHC) staining of the liver sections from different groups of mice as indicated. At 7- day post-infection (peak day of the symptoms), tissues were collected and processed for IHC using anti-VE-Cadherin antibody. The images were viewed under 20X objective.

As observed in the HMEC-1 cell lines (Figure 2(f) and 3(h)), a significant increase in the levels of Src and VE-Cadherin phosphorylation was observed in the liver tissues of dengue-infected mice (Figure 9(a,b)). However, the levels of total VE-Cadherin and total Src remained unchanged. Upon administration of ponatinib, the phosphorylation levels of both proteins returned to their original levels (Figure 9). Immunohistochemical staining of the mouse liver tissue indicated a significant reduction in the sinusoidal expression of VE-Cadherin in DENV2-infected mice whereas in DENV2-infected mice treated with ponatinib, the expression was greatly restored (Figure 9(c)).

### Post-symptom-onset treatment with Ponatinib was effective, but to a lesser extent

**Figure 10.**
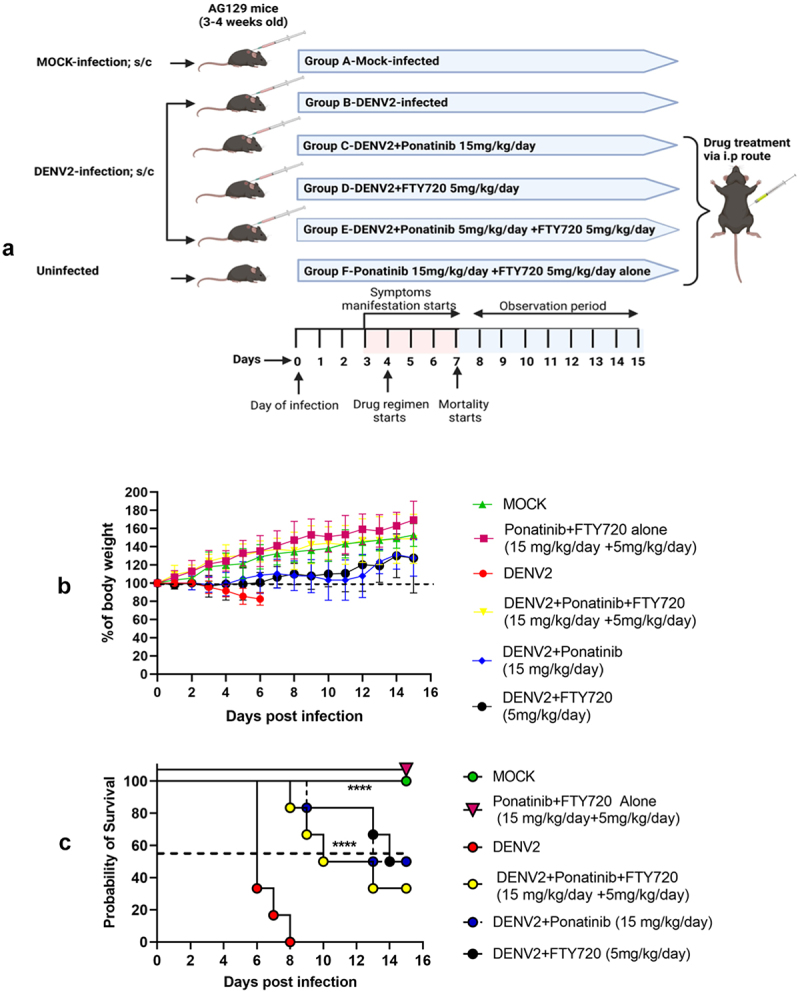
Post-symptom onset treatment with Ponatinib showed comparable improvement in body weight and survivability as with Pre-symptom onset treatment (a) experimental scheme (b) body weight changes expressed as a percentage from the baseline measurement on day 0. The values are mean from 6 animals in each group. (c) Comparative analysis of mortality rates among different groups as mentioned in the scheme. Log-rank (Mantel-Cox) test was used to determine the significance of survival differences based on median survival times. (*N* = 6 for each group).

In another set of experiments in AG129 mice, we evaluated the effect of ponatinib treatment initiation after the onset of clinical symptoms 4^th^ day post-DENV2 infection (Figure 10(a)). We observed that the treatment was effective, but to a lesser extent than the early treatment initiated before the onset of symptoms. The treated animals maintained their body weight and there was no exacerbation of clinical symptoms (Figure 10(b)). On the 10^th^ day of infection in the pre-symptom-onset treatment, 100% of the ponatinib-treated mice were alive, whereas in the post-symptom-onset treatment group, only 80% of the animals were alive (Figure 5(c) and 10(c)). At the end of the 15-day observation period, early treatment resulted in 60% survival in infected mice whereas in the post-symptom-onset treatment group, it was 50% (Figure 5(c) and 10(c)).

Earlier studies from our laboratory established that FTY720, an FDA-approved Sphingosine-1-phosphate (S1P) receptor agonist, significantly improved the survival rates of DENV2-infected AG129 mice [24]. Here also, we observed that FTY720 treatment improved survival rates in infected mice with 50% of the animals surviving at the end of the 15-day observation period (Figure 9(c)). Further, we aimed to evaluate whether post-symptom-onset treatment with a combination of ponatinib (15 mg/kg/day) and FTY720 (5 mg/kg/day) in infected mice could have a synergetic effect on bodyweight and survival rates. The combination treatment was more advantageous in terms of body weight maintenance with animals showing approximately 20–30% higher weight than that of the control infection group throughout the observation period (Figure 10(b)). However, with respect to survival, the combination therapy was inferior, with only 50% alive at 10^th^ day post-infection, and around 30% at 15^th^ day of the observation period against 80% and 50%, respectively, in the treatment with ponatinib alone (Figure 10(c)).

## Discussion

Endothelial cell signalling events play a profound role in altering vascular permeability. In VEGF or in advanced glycation end products (AGE)-induced vascular hyper-permeability, Src-activation and subsequent phosphorylation of VE-Cadherin are central mechanisms [15,30]. Previous studies have also established that selective pharmacological targeting of Src hampers angiogenesis and stabilize endothelial barrier function in cancers [14,31]. Similarly, in rotavirus-induced barrier dysfunction, ROCK-inhibition decreased phosphorylation of myosin light chain (p-MLC2), a downstream target of Rho/ROCK signalling, thereby reducing tight junction permeability [22]. In DENV2-infected HMEC-1 cells, Tie-2 receptor signalling, mediated through Rho/ROCK and Src pathways, plays a key role in VE-Cadherin phosphorylation and internalization, resulting in junctional destabilization [13]. The current study takes these leads further to demonstrate that clinically relevant FDA-approved inhibitors targeting Src-family kinases and RhoA kinases have significant benefits in reducing endothelial permeability in cellular and animal models of DENV2 infection.

Of the five inhibitors used in this study, two ROCK inhibitors, netarsudil and ripasudil, are FDA-approved for use in glaucoma and Amyotrophic Lateral Sclerosis, respectively [27,28,32]. Among the three Src-inhibitors, bosutinib and vandetanib are FDA-approved drugs for use in chronic myelogenous leukaemia and medullary thyroid cancer, respectively [33,34]. Bosutinib has also been shown to reduce Andes virus-induced vascular leakage in human pulmonary endothelial cells [16]. Ponatinib (AP24534), is a novel, potent multi-kinase inhibitor that primarily targets Src kinase activity, and is an FDA approved drug used to treat chronic myelogenous leukaemia (CML), acute myeloid leukaemia (AML) and Philadelphia chromosome – positive (Ph+) acute lymphoblastic leukaemia (ALL) [35–39].

Using the RGCB880/2012 DENV2 strain which enhances monolayer permeability in HMEC-1 cells [23], we observed that treatment with these inhibitors significantly reversed the permeability changes induced by infection. The range of *in vitro* doses of these drugs was determined based on the IC_50_ values reported in the previous studies [26,37,40–43]. To minimize the toxicity, the drugs were used at their lower doses. Furthermore, these compounds were evaluated for their cytotoxicity to HMEC-1 cells as well as their direct antiviral activity against the DENV2 strain used in this study. None of these compounds exerted significant cytotoxicity or anti-DENV2 activity at the doses used in the study (Supplementary Figure S1a,b,c & d). Both TEER measurement and FITC-dextran permeability assays revealed restoration of barrier integrity (Figure 2 and 3). Interestingly, all these specific inhibitors were effective in significantly reducing permeability, albeit at different levels. Src-family kinases (SFKs) are activated by phosphorylation at their tyrosine residue, Y416 [44], which in turn causes VE-Cadherin phosphorylation at Y685 position. In DENV2 infection in HMEC-1 cells, Y416-specific VE-Cadherin phosphorylation was observed [13]. Similarly, inhibition of functional ROCK decreased levels p-MLC2 (Thr18/Ser19) [45]. In our experiments, the increased phosphorylation of VE-Cadherin, MLC2 and Src caused by DENV2-infection could be reduced by the treatment with ponatinib (Figure 2(f) and 3(h)). In addition, immunofluorescence analysis of HMEC-1 cells showed that treatment with Ponatinib restored the membrane- expression of VE-Cadherin that was lost during the DENV2-infection (Figure 4(a,b)). Loss of VE-Cadherin from the cell-membranes upon DENV2 infection was observed both in DENV2-antigen positive cells a well as DENV2-antigen negative cells in the vicinity. This was also observed in our earlier studies [13,24] and could be attributed to the paracrine action of some secreted factors from the infected cells, including the viral protein NS1 [46]. We found that ponatinib treatment restored VE-Cadherin expression in both DENV antigen-positive and -negative cells (Figure 4(b)).

Even though all the inhibitors used in the study showed comparable responses, we performed the *in vivo* experiments with ponatinib, since it inhibited permeability changes at a very low dose (as low as 5 nM) compared to the other compounds tested. In addition, as per the FDA data, the mean half-life of ponatinib is 24 h, which allows once-daily dosing regimen for therapeutic purposes [47]. It was observed that the administration of ponatinib significantly lessens the severity of DENV infection-related complications in the AG129 mice. The ponatinib drug regime started after 1^st^ day post-infection led to a significant improvement in both body weight and survival rates (Figure 5(b,c)). Notably, 100% of the mice treated with ponatinib were alive at day 10 post-infection, while all the DENV2-infected, untreated mice died at this time point. Moreover, 60% of the Ponatinib-treated mice survived on day 15 post-infection, highlighting a substantial improvement in long-term survival.

Ponatinib treatment also had additional benefits as evidenced by the improvement in vascular leakage (Figure 6), leucopenia and thrombocytopenia (Figure 7). Its effectiveness was also observed in reducing the liver enzymes (ALT and AST) that were elevated upon DENV2 infection (Figure 8(a)). It was also seen that in these animals, Ponatinib treatment did not exert any antiviral effect as there was no significant change in the viral titre in whole blood samples (Figure 8(b)). Therefore, it could be presumed that the additional beneficial effects observed upon ponatinib treatment could be due to its immunomodulatory effects, as many tyrosine kinase inhibitors have been attributed to this property [48] in several conditions, including in viral infections such as influenza [49].

Our previous studies demonstrated that DENV2 infection significantly upregulated the transcript levels of proinflammatory cytokines, IL-1β and TNF-α, in virus-infected AG129 mice [25]. Based on these findings, we further assessed the effect of ponatinib on the inflammatory responses in DENV-infection by evaluating the mRNA expression of IL-1β and TNF-α in HMEC-1 cell lysates and in whole blood samples from AG129 mice. *In vitro*, ponatinib treatment did not induce significant changes in the expression of IL-1β or TNF-α in HMEC-1 cells, suggesting that DENV infection or ponatinib treatment may not directly modulate expression of these cytokines in this cell type (Supplementary Figure S3 a & b). On the other hand, *in vivo*, DENV2 infection resulted in a marked upregulation of IL-1β and TNF-α mRNA levels in comparison to mock controls as observed in the earlier studies [25]. Ponatinib treatment in DENV2-infected mice led to a significant reduction in the expression of both IL-1β and TNF-α (Supplementary Figure S3 c & d), indicating that Ponatinib may exert a potential role in modulating inflammatory responses during DENV infection thereby providing an additional benefit in reducing inflammatory cytokine-induced vascular permeability apart from its ability by modulating endothelial signalling pathways.

The disruption of VE-Cadherin in the endothelial cell membrane, which is targeted by multiple signalling pathways, can compromise the endothelial barrier [3,50]. Consistent with the *in vitro* findings (Figure 2 and 3), western blot analysis of liver tissues from dengue-infected and ponatinib-treated mice showed reduced phosphorylation of both Src and VE-Cadherin (Figure 9(a)). Furthermore, immunohistochemical analysis demonstrated that treatment with ponatinib restored sinusoidal VE-Cadherin expression levels in liver tissue of DENV2- infected mice (Figure 9(c)), a finding that was corroborated by immunostaining of VE-Cadherin in HMEC-1 cells (Figure 4(a)).

In clinical settings, early treatment for dengue can be employed only during outbreak conditions, whereas in endemic areas with sporadic dengue cases, patients reach the hospitals only at a later time point from the onset of the disease where the disease gets diagnosed as dengue. Therefore, understanding the effectiveness of a post-symptom onset treatment regimen is beneficial. Accordingly, we treated infected mice with ponatinib after the onset of clinical symptoms. Interestingly, administering ponatinib in this context also, the treatment could impart approximately 50% survival until 15^th^ day post-infection and could be considered a significant improvement in survivability compared to untreated, DENV2-infected controls (Figure 10). However, it was slightly inferior to an early time-point treatment where 60% survivability was observed.

In our previous studies, we demonstrated that FTY720 (Fingolimod), an FDA-approved S1P receptor agonist, reduced VE-Cadherin phosphorylation and DENV2-induced endothelial hyperpermeability, resulting in a 70% survival rate in AG129 mice treated with the drug up to day 10 post-infection [24]. Since both ponatinib and FTY720 mediate their effects through a reduction in VE-Cadherin phosphorylation, we used a combination of them in a post-symptom onset treatment regime, to evaluate any synergism. However, this combination did not show any added protection in terms of survival, even though the animals showed better maintenance of body weight (Figure 10(b)).

One of the limitations of our study was the use of AG129 mice as a model. These mice are immunocompromised and do not fully mimic the complex immune responses and disease manifestations observed in humans. Their lack of interferon signalling alters the immune environment significantly, which may not accurately represent human dengue disease mechanisms [51]. Second, we observed that in animals treated with ponatinib alone, there were changes in haematological parameters, such as leucopenia, lymphopenia and reduction in haematocrit to some extent; however, these changes did not affect their survival for the period of observation in our experiments. (Figure 5 and 7). According to FDA reports, ponatinib treatment is associated with significant adverse effects including vascular occlusive events, hepatoxicity, cardiovascular toxicity and heart failure which are highlighted in boxed warnings, reflecting the serious nature of these potential side effects [47]. Most severe toxic effects occur after extended periods of therapy; for instance, arterial occlusive events typically occur after a median of about 13.4 months of treatment [47,52]. The early adverse effects of ponatinib, such as pancreatitis, typically occur within two weeks (median time to first onset); and are often reversible with appropriate management [36]. In contrast, treatments for plasma leakage in dengue are usually given for a much shorter duration, typically for less than a week, thus posing a significantly reduced risk of severe adverse events.

## Conclusions

In summary, our findings reveal that FDA-approved small molecule inhibitors of Src and Rho kinases effectively mitigate DENV2-induced endothelial hyperpermeability *in vitro* and in animal models. In particular, ponatinib offers substantial therapeutic advantages in the treatment of dengue virus-induced vascular leakage. Moreover, the potential use of these SFK and ROCK inhibitors may be applicable to other viral diseases, such as Zika, Ebola or Hanta virus infections, wherein vascular leakage contributes to disease pathogenesis [53–55]; as well as conditions such as sepsis that affect vascular permeability [56,57]. Non-human primate model-based studies and human clinical trials will effectively translate these observations into clinical settings for patient therapy and management.

## Supplementary Material

Supplementary Fig 2.tif

R1 Supplemenatary Figure Legends Ed.docx

Supplementary Fig 1.tif

Supplementary Fig 3.tif

R1 Supplemenatary Table Ed.docx

2 ARRIVE author check list R1 Ed.pdf

## Data Availability

The authors confirm that the data supporting the findings of this study are available within the article [and/or] its supplementary materials. The study adhered to the ARRIVE guidelines. Compliance checklist to the ARRIVE guidelines and raw data used for generating the graphs and images are deposited in the data base Figshare and are available through the link https://doi.org/10.6084/m9.figshare.27311427
