# Supplementary figures and images for: Ponatinib and other clinically approved inhibitors of Src and Rho-A kinases abrogate dengue virus serotype 2- induced endothelial permeability
Source: Virulence. 2025 Apr 6;16(1):2489751. doi: 10.1080/21505594.2025.2489751 (PMC11980456; doi:10.1080/21505594.2025.2489751)

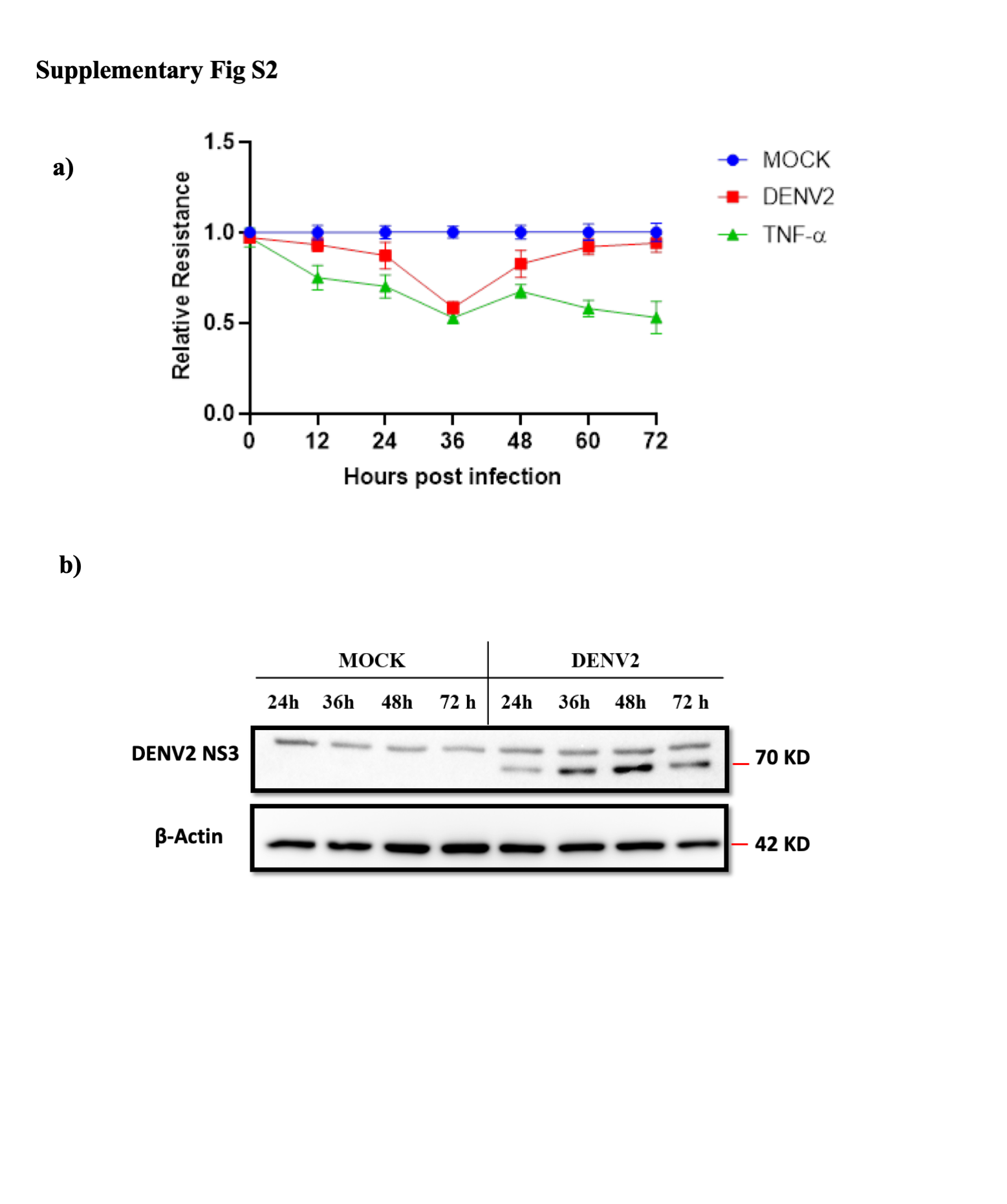

Supplement: Supplementary Fig 2.tif [file KVIR_A_2489751_SM7285.tif]

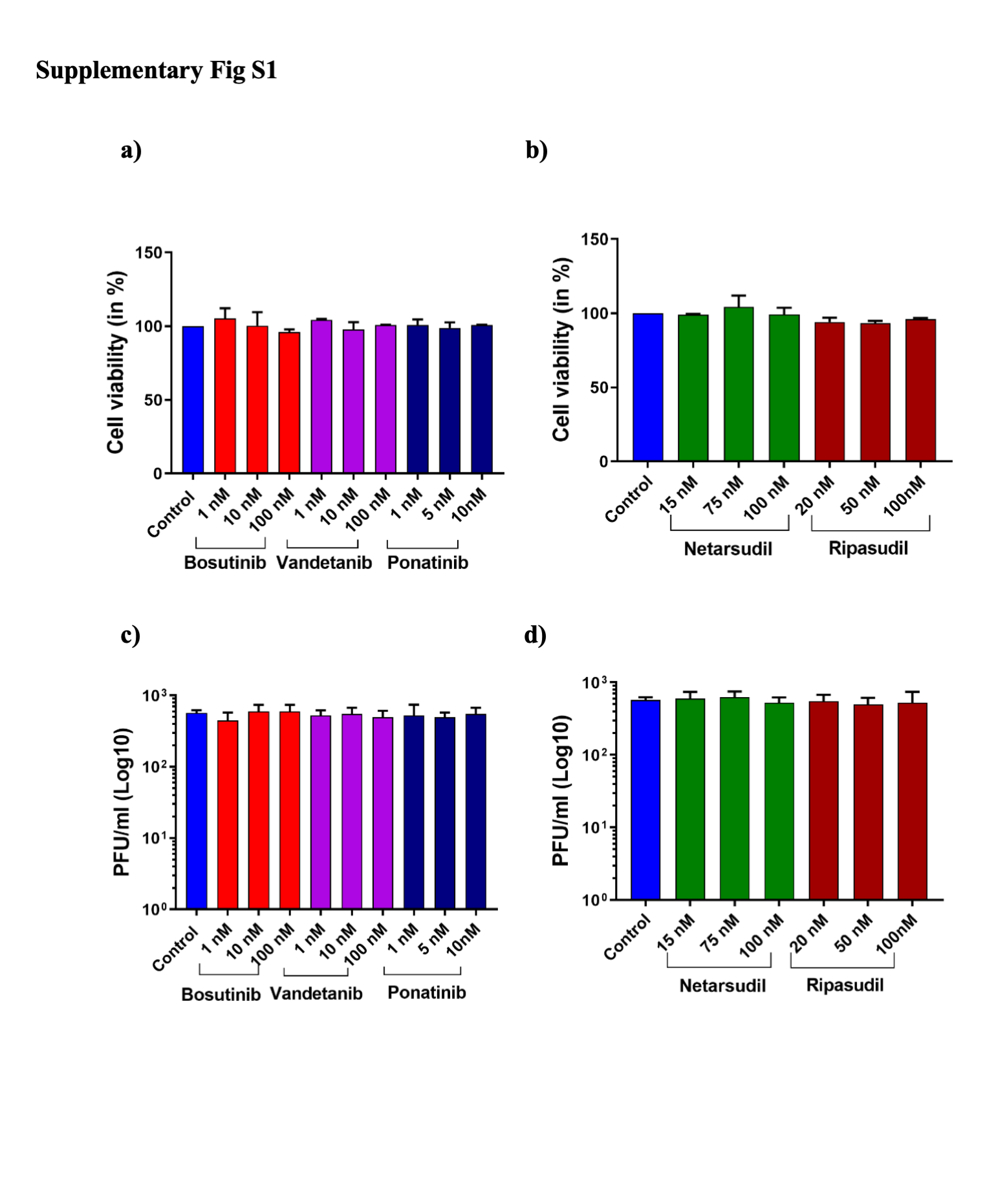

Supplement: Supplementary Fig 1.tif [file KVIR_A_2489751_SM7283.tif]

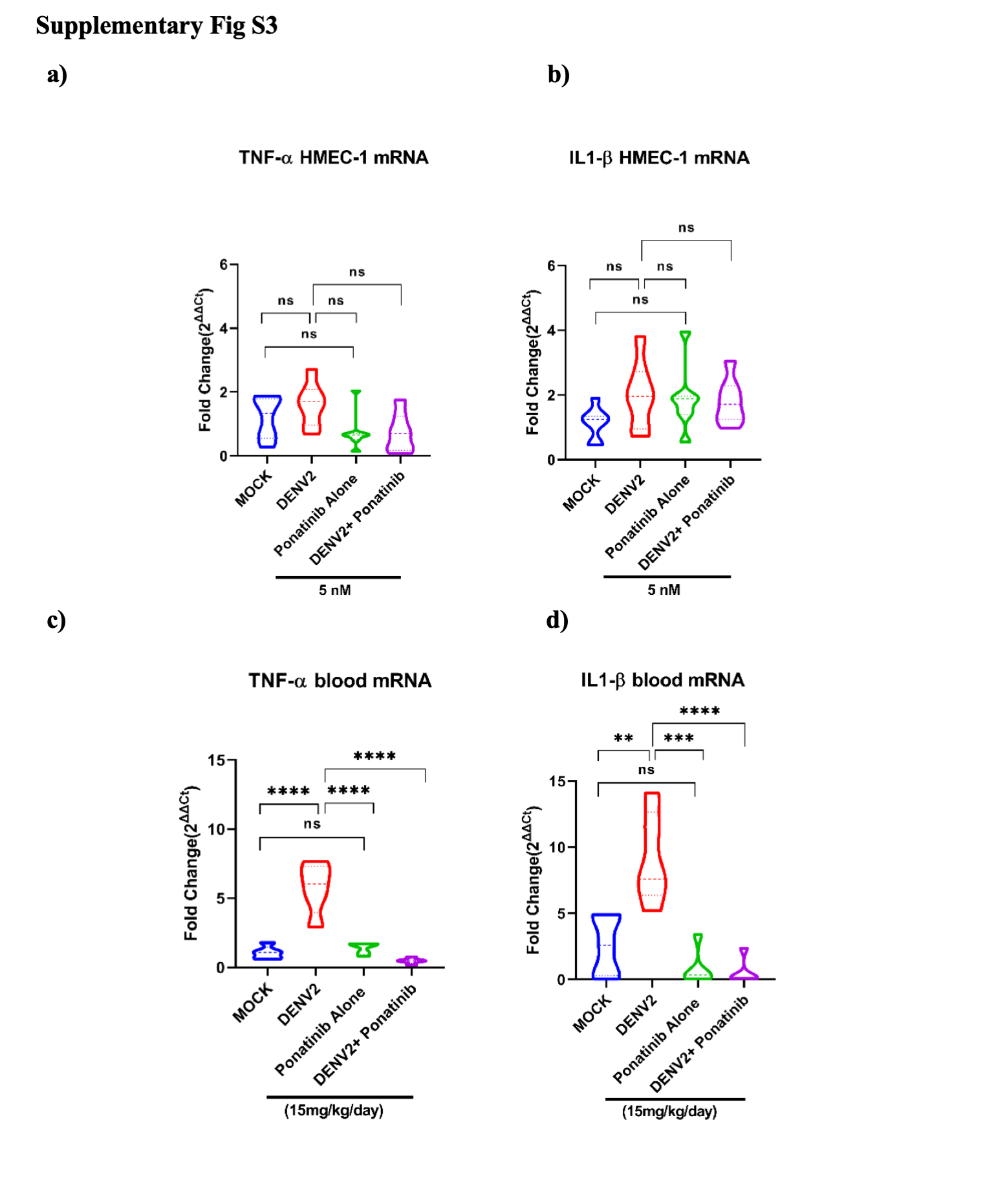

Supplement: Supplementary Fig 3.tif [file KVIR_A_2489751_SM7282.tif]
