# Supplementary material for: Ponatinib and other clinically approved inhibitors of Src and Rho-A kinases abrogate dengue virus serotype 2- induced endothelial permeability
Source: Virulence. 2025 Apr 6;16(1):2489751. doi: 10.1080/21505594.2025.2489751 (PMC11980456; doi:10.1080/21505594.2025.2489751)
Supplement: R1 Supplemenatary Figure Legends Ed.docx [file KVIR_A_2489751_SM7284.docx]

**Supplementary Figure Legends**

**Fig S1. Cytotoxicity evaluation and anti-DENV activity of the test compounds.** Treatment with SFK and ROCK inhibitors does not show toxicity in HMEC-1 cells and does not have anti-viral activity against DENV2 infection. Evaluation of cytotoxicity of SFK (a) and ROCK inhibitors (b) in HMEC-1 cells by MTT assay using different concentrations of drug. Culture supernatants from HMEC-1 cells infected with DENV-2 followed by treatment with SFK (c) and ROCK inhibitors (d) were collected and subjected to plaque assay in BHK-21 cells. Experiments were done in triplicates and mean values are shown.

**Fig S2.** **DENV infection induces endothelial permeability in HMEC-1 cells**. HMEC-1 cells were infected with DENV-2 (RGCB880) at a multiplicity of infection (MOI) 5. (a) Trans-endothelial electrical resistance (TEER) assay to evaluate monolayer permeability. Y-axis represents the relative resistance with respect to mock-infected (heat-inactivated virus) controls. (b) Western blot for DENV NS3 protein expression in HMEC-1 cells. Time kinetics analysis was carried out to detect the DENV2 NS3 expression. Expression of β-Actin is shown as the loading control.

**Fig S3.** **Ponatinib treatment suppresses DENV2-induced pro-inflammatory cytokine response in AG129 mice.** HMEC-1 cells or AG129 mice were infected with DENV2 followed by Ponatinib treatment. a) & b) Transcript levels of TNF-α and IL-1β in HMEC-1 cells collected 36 h.p.i; or c) & d) in whole blood collected on day 6 p.i. in DENV2-infected mice. Total RNA was isolated from both HMEC-1 cells or whole blood in mice and relative quantification of the transcripts by real-time PCR was done with gene-specific primers as described previously [25]. The values were normalized against β-actin transcript levels of the respective samples. Fold-change was calculated using the 2^−∆∆Ct^ method. For *in-vitro* analysis, mean values are represented from three independent experiments, each with duplicate readings. (N=6). For *in-vivo* analysis, each data value represents the average of triplicate readings from an individual animal. Average values from five mice per group is plotted in the graph (N=5). Statistical analysis was done by one-way ANOVA with Sidak's multiple comparisons test. Significance is denoted by “*” (p<0.05), “**” (p<0.005), “***” (p<0.0005), “****” (p<0.00005), and “ns” for non-significant results.
