# Supplementary material for: Ponatinib and other clinically approved inhibitors of Src and Rho-A kinases abrogate dengue virus serotype 2- induced endothelial permeability
Source: Virulence. 2025 Apr 6;16(1):2489751. doi: 10.1080/21505594.2025.2489751 (PMC11980456; doi:10.1080/21505594.2025.2489751)
Supplement: R1 Supplemenatary Table Ed.docx [file KVIR_A_2489751_SM7281.docx]

**Supplementary Table**

**Table S1**. List of antibodies used in this study:

| **Antibodies** | **Catalogue No. and Source** |
| --- | --- |
| **1)Primary Antibodies:** |  |
| Goat anti-VE-Cadherin | sc-6458; Santa Cruz Biotechnology, USA |
| Rabbit anti-phospho-VE-Cadherin (Y685) | ab119785; Abcam |
| Mouse anti-DENV2 NS3 | SAB2702245; SIGMA, USA |
| Rabbit anti-MLC2 (myosin light chain 2) | 8505; Cell Signaling Technology, USA |
| Rabbit anti-phospho-MLC2 (Ser 18/Thr 19) | 3674; Cell Signaling Technology, USA |
| Rabbit anti-Src | 2109; Cell Signaling Technology, USA |
| Rabbit anti-phospho-Src (Tyr 416) | 6943; Cell Signaling Technology, USA |
| Rabbit anti-β-actin | 4967; Cell Signaling Technology, USA |
| Mouse anti-dengue E glycoprotein antibody | ab41349; Abcam |
|  |  |
|  |  |
| **2)Secondary Antibodies:** |  |
| Anti-rabbit IgG, HRP-linked Antibody | 7074; Cell Signaling Technology, USA |
| Anti-mouse IgG, HRP-linked Antibody | 7076; Cell Signaling Technology, USA |
| Rabbit anti-goat IgG-HRP | sc-2768; Santa Cruz Biotechnology, USA |
| Anti-mouse Alexa fluor 488 | A1008; Invitrogen, USA |
| Anti-rabbit Alexa Fluor 647 | 821246; Invitrogen, USA |
|  |  |
